# External validation of a tumor growth inhibition-overall survival model in non-small-cell lung cancer based on atezolizumab studies using alectinib data

**DOI:** 10.1007/s00280-023-04558-z

**Published:** 2023-07-06

**Authors:** Nastya Kassir, Phyllis Chan, Steve Dang, René Bruno

**Affiliations:** 1 Genentech, Inc., 1 DNA Way, South San Francisco, CA USA; 2 Genentech/Roche, Marseille, France

**Keywords:** Pharmacometrics, Oncology, Survival analysis

## Abstract

**Background:**

A modeling framework was previously developed to simulate overall survival (OS) using tumor growth inhibition (TGI) data from six randomized phase 2/3 atezolizumab monotherapy or combination studies in non-small-cell lung cancer (NSCLC). We aimed to externally validate this framework to simulate OS in patients with treatment-naive advanced anaplastic lymphoma kinase (ALK)-positive NSCLC in the alectinib ALEX study.

**Methods:**

TGI metrics were estimated from a biexponential model using longitudinal tumor size data from a Phase 3 study evaluating alectinib compared with crizotinib in patients with treatment-naive ALK-positive advanced NSCLC. Baseline prognostic factors and TGI metric estimates were used to predict OS.

**Results:**

286 patients were evaluable (at least baseline and one post-baseline tumor size measurements) out of 303 (94%) followed for up to 5 years (cut-off: 29 November 2019). The tumor growth rate estimate and baseline prognostic factors (inflammatory status, tumor burden, Eastern Cooperative Oncology Group performance status, race, line of therapy, and sex) were used to simulate OS in ALEX study. Observed survival distributions for alectinib and crizotinib were within model 95% prediction intervals (PI) for approximately 2 years. Predicted hazard ratio (HR) between alectinib and crizotinib was in agreement with the observed HR (predicted HR 0.612, 95% PI 0.480–0.770 vs. 0.625 observed HR).

**Conclusion:**

The TGI-OS model based on unselected or PD-L1 selected NSCLC patients included in atezolizumab trials is externally validated to predict treatment effect (HR) in a biomarker-selected (ALK-positive) population included in alectinib ALEX trial suggesting that TGI-OS models may be treatment independent.

**Supplementary Information:**

The online version contains supplementary material available at 10.1007/s00280-023-04558-z.

## Introduction

Alectinib is a highly selective anaplastic lymphoma kinase (ALK) tyrosine kinase inhibitor. Alectinib is a preferred first-line therapy for patients with advanced ALK-positive non-small-cell lung cancer (NSCLC) [1, 2]. The randomized, multicenter, open-label, global phase 3 ALEX study compared the efficacy and safety of alectinib with crizotinib in patients with advanced ALK-positive NSCLC [3]. The investigator-assessed progression-free survival (PFS) data (cut-off: 30 November 2018) showed significantly prolonged PFS with alectinib compared to crizotinib [median PFS 34.8 versus 10.9 months crizotinib; hazard ratio (HR) 0.43, 95% confidence interval (CI) 0.32–0.58]. However, the overall survival (OS) data up to 5 years (data cut-off: 29 November 2019) remain immature for alectinib with 37% of events recorded (stratified HR 0.67, 95% CI 0.46–0.98). Median OS was not reached (NR) with alectinib and was 57.4 months with crizotinib (95% CI 34.6–NR) [4].

In recent years, the use of tumor dynamic modeling to support drug development and early decisions has significantly increased by drug developers and regulatory agencies [5–7]. Model-based tumor dynamics metrics (including early shrinkage, time to regrowth, on-treatment growth rate, or the full dynamic profile) have been shown to predict overall survival (OS) in different types of solid tumors, including NSCLC for a variety of treatments [7–9]. A recent review can be seen in Bruno et al. [6]. A modeling framework was previously developed to simulate overall survival (OS) using tumor growth inhibition (TGI) data from six phase 2/3 atezolizumab-containing studies in NSCLC [10]. This model will be referred to herein as the “historical” model. This model included on-treatment tumor growth constant (KG) and independent baseline prognostic factors to predict the OS distributions and hazard ratio.

The objective of our analysis was to externally validate this framework to simulate OS and more specifically alectinib vs. crizotinib treatment effect (OS HR) in patients with treatment-naive advanced ALK-positive NSCLC in the alectinib ALEX study [4].

## Materials and methods

The ALEX study design has been published previously [3]. Briefly, patients aged ≥ 18 years with previously untreated stage III/IV ALK-positive NSCLC were randomized 1:1 to crizotinib (250 mg BID) or alectinib (600 mg BID). Randomization was stratified according to ECOG performance status (0/1 versus 2), race (Asian versus non-Asian) and baseline central nervous system metastases (present versus absent). Patients were treated until disease progression, unacceptable toxicity, withdrawal of consent, or death, whichever occurs first. The trial was approved by the institutional review board or independent ethics committee. All patients provided written informed consent. All authors had access to the study data and reviewed and approved the final manuscript.

Tumor assessment consisted at minimum of a computed tomography or magnetic resonance imaging scan at baseline. Clinical lesions were assessed with caliper measurement and documented by color photography, including a ruler to estimate the size of the lesion. Tumor assessments were carried out in all patients at baseline and every 8 weeks until disease progression or death. Longitudinal tumor size data, defined as the sum of the longest diameters (SLD) of target lesions at each visit according to RECIST 1.1, were used for the estimation of TGI metrics. Patients with at least baseline and one post-baseline tumor size measurements were defined as evaluable, and data from patients who only had baseline tumor assessments were excluded from the analysis.

The previously published biexponential TGI [11] model implemented as a population model as in Claret et al. [9], was fit to the longitudinal tumor size data from ALEX study:

$$TS\left( t \right) = \left\{ {\begin{array}{*{20}c} {TS_{0} \cdot \exp \left( {KG \cdot t} \right)} & {if\;t < 0} \\ {TS_{0} \cdot \left[ {\exp \left( { - KS \cdot t} \right) + \exp \left( {KG \cdot t} \right) - 1} \right]} & {if\;t \ge 0} \\ \end{array} } \right.$$

where *t*: time (week); TS: tumor size (SLD in mm), TS_0_: model-estimated TS at time 0 (treatment start), KG: tumor growth rate constant (week^−1^); KS: tumor shrinkage rate constant (week^−1^).

The model was implemented as a nonlinear mixed-effect model [9] using NONMEM version 7.4. The inter-individual variability of KG and KS by treatment was characterized by a log-normal distribution, with a common log-normal distribution for TS_0_. The additive residual error was described by a normal distribution. TGI model evaluation was based on the inspection of goodness of fit plots. Individual post hoc estimates of KG based on ALEX SLD data were used as TGI metrics in the subsequent TGI-OS modeling.

The OS data from ALEX study were used to externally validate the historical TGI-OS model developed across all atezolizumab NSCLC studies [10]. The historical TGI-OS model was re-run and the parameter estimates were updated using atezolizumab data to only consider covariates available or relevant in ALEX study. 1000 replicates of the Alex study were simulated using the updated historical model parameter estimates. OS distributions, survival rate (% of patients surviving) at select time points, and HR of alectinib versus crizotinib and their 95% prediction intervals (PI) using baseline covariates of ALEX patients and individual KG (based on ALEX SLD data) were obtained. Model parameters were sampled from the estimated mean values and uncertainty in parameter estimates for each of the simulated study replicate. Censoring was simulated by sampling patient study duration from a uniform distribution based on observed censoring. The performance of the TGI-OS model in NSCLC was evaluated by comparing predicted treatment effect (HR) with the observed HR.

## Results

The ALEX patient population has been previously described [3]. A total of 303 patients were randomized to receive treatment [*n* = 152, alectinib; *n* = 151, crizotinib; intent-to-treat (ITT) population].

A total of 286 (94%) out of 303 treated patients in ALEX study were defined as TGI evaluable [cut-off: 29 November 2019]. Descriptive statistics of the baseline prognostic factors of the TGI-evaluable patients from the ALEX trial and from the atezolizumab first-line NSCLC trials are presented in Supplementary Table S1 and Table S2, respectively. A total of 4003 tumor assessments were used for the TGI analysis with a median (range) follow-up time of 79.6 (0.14–271.3) weeks. The TGI model described ALEX tumor size profiles well with high precision (< 20%) on parameter estimates (Table 1) and good correlation between observed and predicted SLD (Fig. S1). The low shrinkage on the inter-individual variability of KG indicates that individual post hoc estimates can be used reliably in the TGI-OS simulations. An example of individual fits is presented in Fig. S2. Of interest, estimated KG in the alectinib arm was much slower than in the crizotinib one: 0.00196 vs. 0.00438 1/week while the estimated KS were similar.

**Table 1:** Parameter estimates from tumor growth inhibition model

	Estimate [ϴ]	RSE (%)	Estimate [ω^2^] (IIV CV(%))	RSE (%)	%Shrinkage
KG_alectinib_ (1/week)	0.00196	13.2	1.90 (138)	17.0	8.8
KS_alectinib_ (1/week)	0.0342	9.8	0.943 (97.1)	11.8	11.3
KG_crizotinib_ (1/week)	0.00438	12.1	1.10 (105)	20.8	9.2
KS_crizotinib_ (1/week)	0.0373	10.5	0.833 (91.3)	16.0	13.7
TS_0_ (mm)	57.0	4.2	0.436 (66.1)	8.4	4.9
σ^2^ (mm)	42.5	13.4			

*CV* coefficient of variation, *IIV* inter-individual variability, *KG* tumor growth rate constant (1/week), *KS* tumor shrinkage rate constant (1/week), *RSE* relative standard error of parameter estimate, *TS* tumor size (SLD in mm), *TS*_*0*_ TS at time 0 (treatment start), *ϴ* parameter estimate, *σ*^*2*^ variance of residual error, *ω*^*2*^ variance of inter-individual variability

This historical model included on-treatment tumor growth constant (KG) and independent prognostic factors such as baseline albumin (ALB), C-reactive protein (CRP), lactate dehydrogenase (LDH), neutrophil-to-lymphocyte ratio (NLR), Eastern Cooperative Oncology Group performance status (ECOG), race (Asian vs. non-Asian), presence of liver metastases, and PD-L1 expression (IC or TC > 0). The parameters in the historical model were re-estimated using the atezolizumab study data [10] to only consider covariates available in ALEX (i.e., CRP and LDH were not available in ALEX). In addition, the effect of IC/TC PD-L1 expression was removed from the model as it is specific to atezolizumab or anti-PD-1/PD-L1 agents and was not available in ALEX. The updated model included the effect of sex and baseline SLD in addition to the previously identified covariates. Parameter estimates from the updated model are presented in Table 2. A comparison of parameter estimates from the historical and the updated models is presented in Supplementary Table S3. The updated model indicates an increase in survival probability for lower KG, baseline SLD, NLR, number of metastatic sites and higher albumin. The model also indicates an increase in survival probability for patients with ECOG of 0 (versus ≥ 1), Asian (versus non-Asian), absence of liver metastasis, first-line of therapy (versus 2 +), or female.

**Table 2:** Parameter estimates from TGI-OS model in atezolizumab NSCLC studies

Parameter	Estimate	SE	*z*	*p*
Intercept	2.87	0.164	17.50	< 2e–−16
logKG	− 0.642	0.0224	− 28.62	< 2e−16
Albumin (g/L)	0.0262	0.00274	9.53	< 2e−16
ECOG PS (≥ 1 vs. 0)	− 0.270	0.0295	− 9.12	< 2e−16
Race (Asian vs. non-Asian)	− 0.319	0.0433	7.36	1.90E−13
Number of metastatic sites	− 0.0733	0.0147	− 4.97	6.70E−07
Neutrophil-to-lymphocyte ratio	− 0.0138	0.00263	− 5.25	1.50E−07
Liver metastasis (yes vs. no)	− 0.174	0.0396	− 4.39	1.20E−05
Baseline SLD (mm)	− 0 .00122	0.000318	− 3.82	0.00013
Line of therapy (2 + vs. 1)	− 0.103	0.0346	− 2.97	0.003
Sex (female vs. Male)	0.0840	0.0300	2.80	0.00512
Log(scale)	− 0.225	0.0155	− 14.51	< 2e−16

Survival time was analyzed in days

*ECOG* Eastern Cooperative Oncology Group performance status (reference group is 0), *log(KG)* log of tumor growth rate constant (1/week) from the tumor growth inhibition model; *p* Wald test *p* value, *scale* standard deviation of log (OS), *SE* standard error of parameter estimate, *SLD* sum of longest diameter, *z* Wald statistic

^a^Number of metastatic sites was equal to 5 if the number is  ≥ 5, otherwise it is equal to the number of metastatic sites

**Fig. 1:**
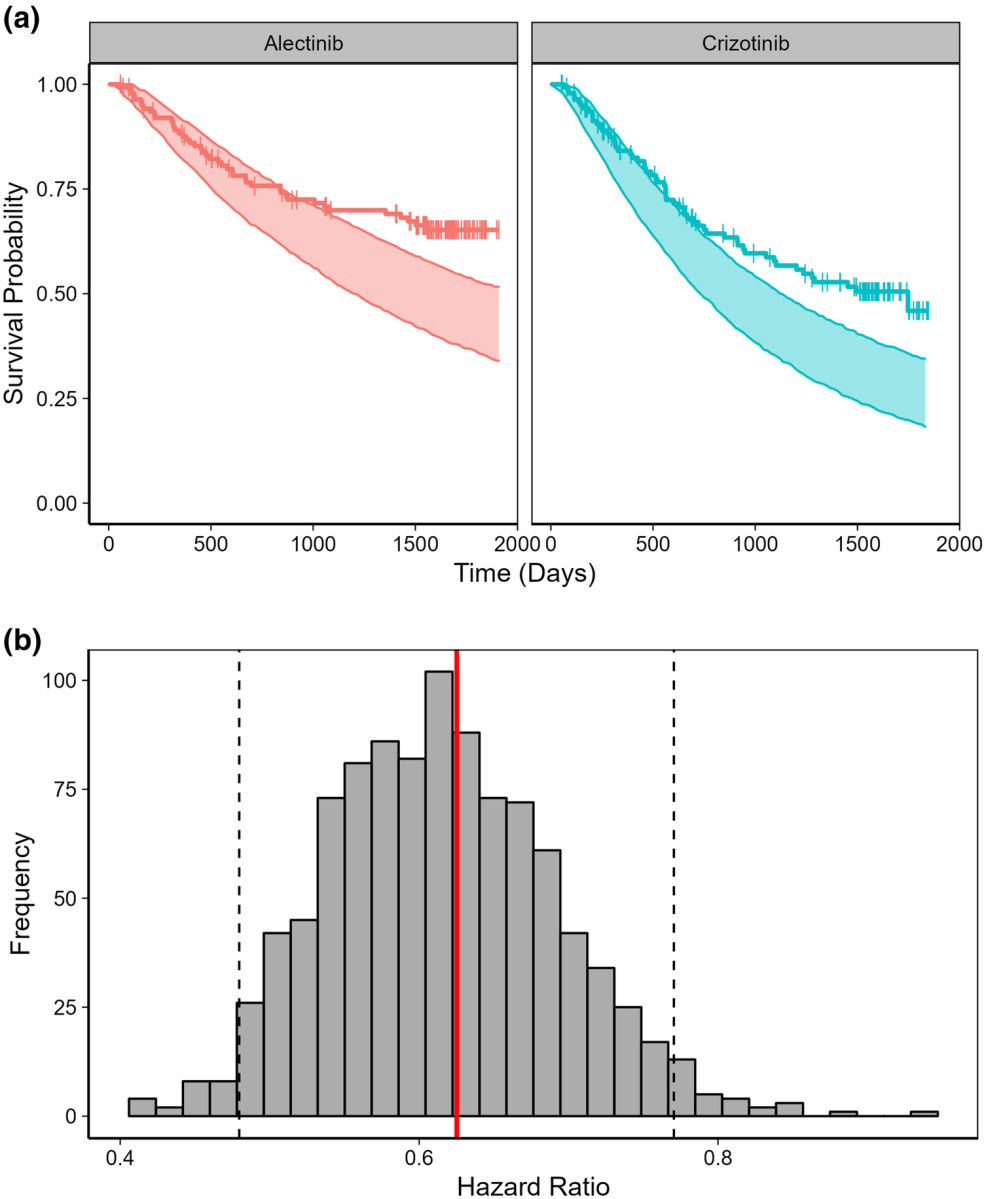
Predicted and observed **A** OS distributions and **B** hazard ratios in the ALEX study. **a** Lines: Observed survival; Vertical: censored data; Areas: 95% prediction intervals; **b** Red line: Observed hazard ratio; Dashed lines: 95% prediction intervals

Simulated survival distributions (median and 95% PI) were first compared to observed distributions of alectinib and crizotinib. As can be seen in Fig. 1a, the simulated distributions captured the observed ones for the first 2 years of treatment after what observed OS was longer than predicted for both arms. The OS data were not mature with the median OS was not reached in the alectinib arm and under-predicted by the model in the crizotinib one. The observed 2 years survival rate for alectinib (75.8%) was within model 95% PI [72.4 (65.7, 79.0)] while for crizotinib it was slightly outside: 66.2% vs. 57.4% (49.9, 64.7).

On the other hand, the observed HR between alectinib and crizotinib was within model-prediction interval: 0.625 vs. 0.612 (95% PI 0.480–0.770) (Fig. 1b). Note that the upper bound of the 95% PI is below 1, consistent with an improvement of OS with alectinib compared with crizotinib.

## Discussion

Clinical oncology drug development remains challenging with both a need for new efficacious and safe drugs and also identification of markers of efficacy to support early development decisions. Overall survival is considered the most reliable efficacy endpoint in oncology but could take years to be available to mature. Different models have been developed to characterize the relationship between early biomarkers and overall survival. The value of TGI parameters to predict overall survival have been demonstrated in different types of solid tumors. These TGI parameters encompass early shrinkage, time to regrowth, on-treatment growth rate, or the full dynamic profile. By leveraging TGI-OS models, several potential applications arise, including aiding early decisions between treatments, identifying promising combinations for future clinical trials, and assessing the likelihood of success for phase III trials [6, 12, 13]. In a recent publication, we examined the operating characteristics of TGI metrics on artificially truncated data derived from the successful Phase III study of atezolizumab (IMpower150). This evaluation aimed to replicate early findings from Phase Ib/II studies, where patient numbers were small, and follow-up was limited. Our analysis revealed that TGI metrics exhibited superior performance in distinguishing between Phase III success and failure compared to ORR or PFS [14].

Currently, these models are used in the same patient population and tumor type from which they were developed. In order to get confidence with applying tumor dynamic based decision frameworks to support early decisions, it is critical to perform external validation using studies with different treatment and populations [15]. The objective of this analysis was to externally validate the TGI-OS model based on unselected or PD-L1 selected NSCLC patients included in atezolizumab trials to predict treatment effect (HR) in a biomarker-selected (ALK-positive) population included in alectinib ALEX trial. In the historical model, KG was the most influential TGI metric, selected based on the data from six studies investigating atezolizumab, a monoclonal antibody.

Due to the differences in data availability between clinical trials, the historical model was updated to only include eight out of the 11 covariates in the historical model that were also available in the ALEX trial dataset and tested for additional covariates. After removal of CRP, LDH, and IC/TC PD-L1, the additional covariates identified were baseline SLD and sex. The directions of the baseline prognostic factor effects were the same between the historical and the updated models for the eight covariates that are common in the two models, and the effects remained statistically significant (p value < 0.01) for all nine covariates. The change in the parameter estimates of the nine covariates ranged from -5.50% (line of therapy) to + 94.1% (ALB). It is likely that the effects in the historical model from CRP and LDH, which were not available in the ALEX trial dataset, were re-distributed to ALB in the updated model. Therefore, a large change in the estimated ALB effect was observed. In the updated model, KG remained the most statistically significant covariate out of the 10 estimated ones which is consistent with results reported in immune checkpoint inhibitors or targeted therapies [12].

The ALEX trial data consisted of treatment-naïve patients with ALK-positive NCSLC (*N* = 286 TGI evaluable). The survival distributions for alectinib and crizotinib were within model-prediction intervals for ~ 2 years out of the 5 years of follow-up. The difference in survival plots between simulation and ALEX could be because the OS data is immature. However, it cannot be ruled out that ALK-positive patients may also have distinct disease features that are not captured by the model based on atezolizumab data. Indeed OS is much longer in this first-line NSCLC population (e.g., median of 57 months for crizotinib and not reached for alectinib) compared to the typical OS in the atezolizumab studies of 15–20 months that were used to train the model. ALK-positive first-line NSCLC patients were preferably treated by an ALK-TK inhibitor. However, the predicted HR between arms was in agreement with the observed HR indicating that the difference in estimated KG between arms translated into a difference in OS and the model was able to predict alectinib treatment benefit even though the model under-predicted OS distributions.

The predictability of KG in the historical model was confirmed for small molecule tyrosine kinase inhibitor therapy in a biomarker-selected first-line NSCLC population using ALEX trial data. This supports the use of a TGI metrics like KG to support early decisions [14] in combination with small molecules as well as monoclonal antibodies with novel mechanism of actions. The fact that the model was unable to predict long-term OS distributions is clearly a limitation that will need to be addressed with more data in this ALK-positive patients populations. More experience needs be accrued about the applicability of the (historical or updated) models with other classes or modalities of cancer treatments (e.g., such as vaccines). Other longitudinal biomarkers like inflammatory markers or more mechanistic ones like ctDNA are currently being explored [15].

## Supplementary Information

Below is the link to the electronic supplementary material.

Supplementary file 1 Fig. S1 Goodness of fit plots of the TGI model using ALEX data. I.: Individual; TGI: tumor growth inhibition (TIFF 10454 KB)

Supplementary file 2 Fig. S2 Sample individual TGI model fits from ALEX patients. TGI: tumor growth inhibition (TIFF 10454 KB)

Supplementary file3 (DOCX 14 KB)

Supplementary file4 (DOCX 16 KB)

Supplementary file5 (DOCX 14 KB)

## Data Availability

Data availability statement Qualified researchers may request access to individual patient-level data through the clinical study data request platform (https://vivli.org/). Further details on Roche’s criteria for eligible studies are available at https://vivli.org/members/ourmembers/. For further details on Roche’s Global Policy on the Sharing of Clinical Information and how to request access to related clinical study documents, see https://www.roche.com/research_and_development/who_we_are_how_we_work/clinical_trials/our_commitment_to_data_sharing.htm.

## Declarations

### Statements and declarations

The authors are employees and stockholders of Genentech/Roche.
