# Supplementary figures and images for: External validation of a tumor growth inhibition-overall survival model in non-small-cell lung cancer based on atezolizumab studies using alectinib data
Source: Cancer Chemother Pharmacol. 2023 Jul 6;92(3):205–10. doi: 10.1007/s00280-023-04558-z (PMC10363035; doi:10.1007/s00280-023-04558-z)

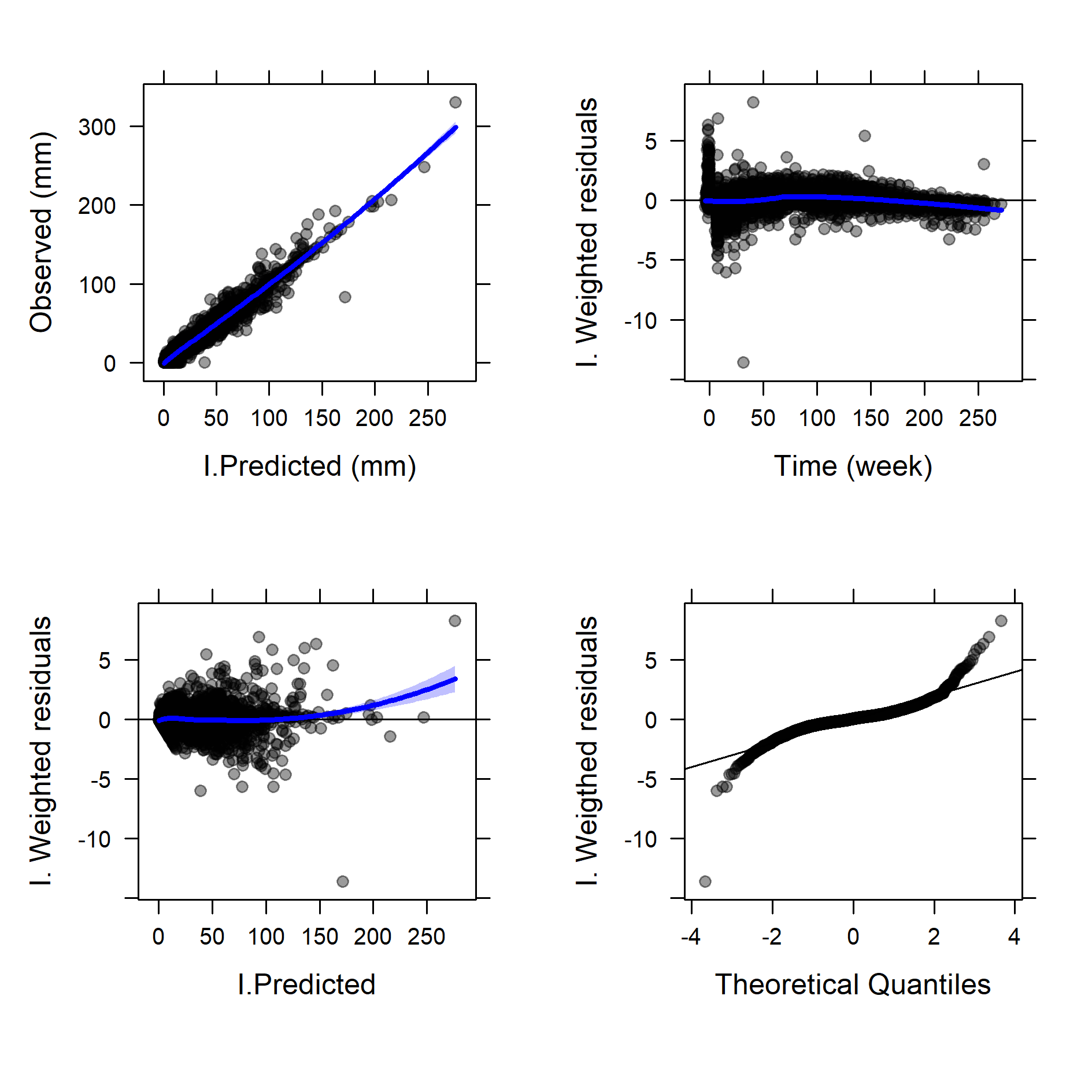

Supplement: Supplementary file 1 — Fig. S1 Goodness of fit plots of the TGI model using ALEX data. I.: Individual; TGI: tumor growth inhibition (TIFF 10454 KB) [file 280_2023_4558_MOESM1_ESM.tiff]

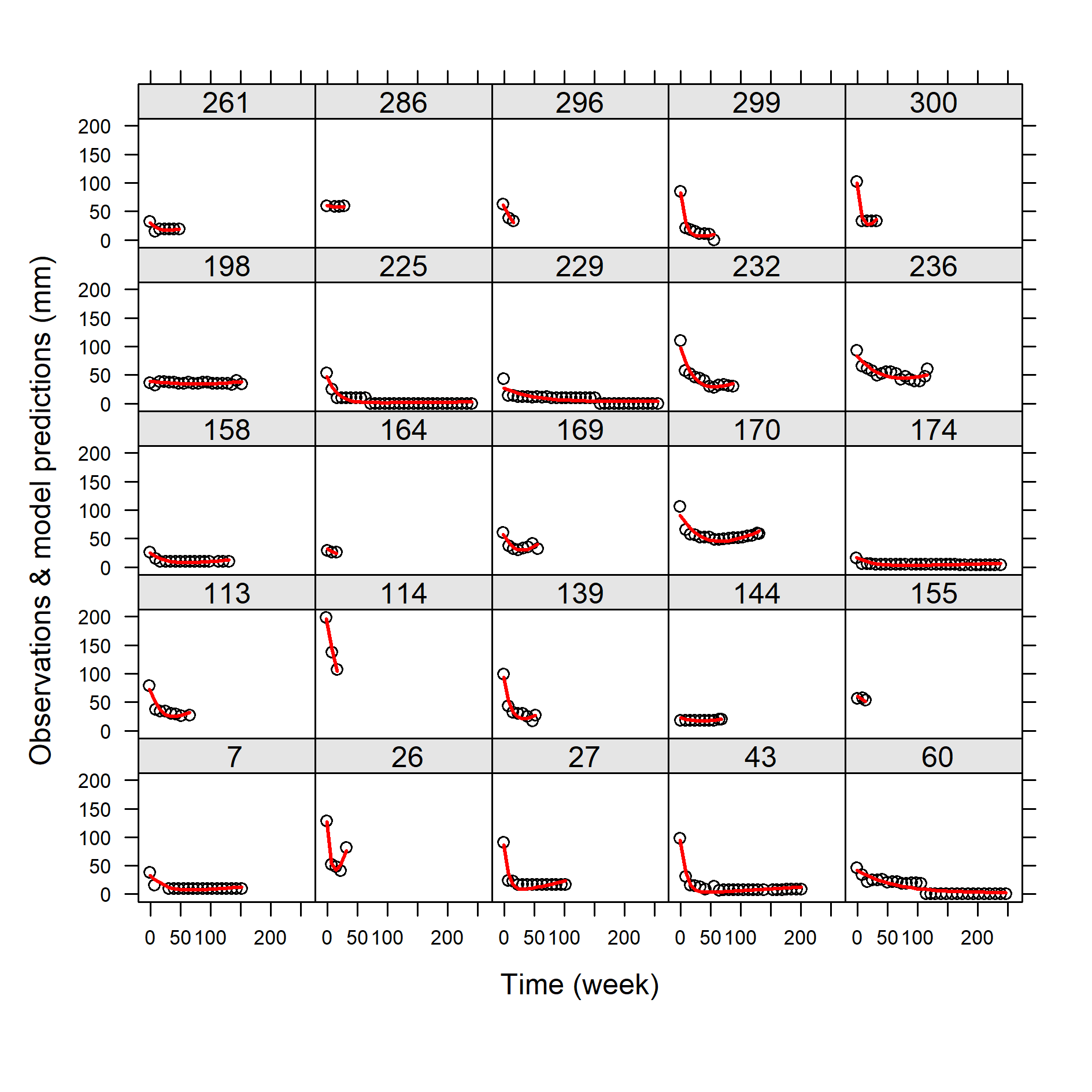

Supplement: Supplementary file 2 — Fig. S2 Sample individual TGI model fits from ALEX patients. TGI: tumor growth inhibition (TIFF 10454 KB) [file 280_2023_4558_MOESM2_ESM.tiff]
