# Supplementary material for: External validation of a tumor growth inhibition-overall survival model in non-small-cell lung cancer based on atezolizumab studies using alectinib data
Source: Cancer Chemother Pharmacol. 2023 Jul 6;92(3):205–10. doi: 10.1007/s00280-023-04558-z (PMC10363035; doi:10.1007/s00280-023-04558-z)
Supplement: Supplementary file 3 — Supplementary file3 (DOCX 14 KB) [file 280_2023_4558_MOESM3_ESM.docx]

**Table S1** Summary statistics of baseline prognostic factors in the tumor growth inhibition-overall survival model from the ALEX trial

| **Covariates** | **ALEX (N=286)** |
| --- | --- |
| **Tumor growth rate (1/week)** | |
| Mean (SD) | 0.00627 (0.00876) |
| Median [Min, Max] | 0.00352 [0.0000509, 0.0631] |
| **ECOG performance status** | |
| 0 | 95 (33.2%) |
| 1 | 175 (61.2%) |
| Missing | 16 (5.6%) |
| **Number of metastatic sites** | |
| 1 | 29 (10.1%) |
| 2 | 89 (31.1%) |
| 3 | 95 (33.2%) |
| 4 | 47 (16.4%) |
| 5+ | 26 (9.1%) |
| **Asian** |  |
| No | 153 (53.5%) |
| Yes | 133 (46.5%) |
| **Albumin (g/L)** | |
| Mean (SD) | 39.3 (5.05) |
| Median [Min, Max] | 40.0 [22.0, 53.5] |
| Missing | 1 (0.3%) |
| **Neutrophil-to-lymphocyte ratio** | |
| Mean (SD) | 4.39 (3.22) |
| Median [Min, Max] | 3.47 [0.714, 29.3] |
| Missing | 1 (0.3%) |
| **Liver Metastasis** | |
| No | 217 (75.9%) |
| Yes | 69 (24.1%) |
| **Sum of longest diameter (mm)** | |
| Mean (SD) | 74.8 (43.6) |
| Median [Min, Max] | 68.0 [10.1, 206] |
| **Sex** |  |
| Female | 162 (56.6%) |
| Male | 124 (43.4%) |

Note: ECOG=Eastern Cooperative Oncology Group, N: number of patients; min: minimum; max: maximum; SD: standard deviation.
