# Supplementary material for: External validation of a tumor growth inhibition-overall survival model in non-small-cell lung cancer based on atezolizumab studies using alectinib data
Source: Cancer Chemother Pharmacol. 2023 Jul 6;92(3):205–10. doi: 10.1007/s00280-023-04558-z (PMC10363035; doi:10.1007/s00280-023-04558-z)
Supplement: Supplementary file 4 — Supplementary file4 (DOCX 16 KB) [file 280_2023_4558_MOESM4_ESM.docx]

**Table S2** Summary statistics of baseline prognostic factors in the tumor growth inhibition-overall survival model from the first line non-small cell lung cancer atezolizumab trials

| **Covariates** | **IMpower130 (N=655)** | **IMpower131 (N=628)** | **IMpower132 (N=480)** | **IMpower150 (N=1106)** | **Overall (N=2869)** |
| --- | --- | --- | --- | --- | --- |
| **Tumor growth rate (1/week)** | |  |  |  |  |
| Mean (SD) | 0.0129 (0.0103) | 0.0140 (0.00959) | 0.0150 (0.0106) | 0.0124 (0.0102) | 0.0133 (0.0102) |
| Median [Min, Max] | 0.0102 [0.000594, 0.0915] | 0.0118 [0.000299, 0.0740] | 0.0126 [0.00237, 0.124] | 0.0102 [0.000725, 0.152] | 0.0108 [0.000299, 0.152] |
| **C-reactive protein (mg/L)** | |  |  |  |  |
| Mean (SD) | 35.9 (47.9) | 42.5 (47.6) | 29.3 (40.3) | 30.3 (44.4) | 34.1 (45.6) |
| Median [Min, Max] | 16.1 [0.200, 314] | 25.9 [0.460, 285] | 13.2 [0.230, 265] | 11.3 [0.220, 318] | 15.5 [0.200, 318] |
| Missing | 27 (4.1%) | 23 (3.7%) | 36 (7.5%) | 34 (3.1%) | 120 (4.2%) |
| **ECOG performance status** | |  |  |  |  |
| 0 | 281 (42.9%) | 219 (34.9%) | 196 (40.8%) | 494 (44.7%) | 1190 (41.5%) |
| 1 | 373 (56.9%) | 407 (64.8%) | 284 (59.2%) | 606 (54.8%) | 1670 (58.2%) |
| Missing | 1 (0.2%) | 2 (0.3%) | 0 (0%) | 6 (0.5%) | 9 (0.3%) |
| **Number of metastatic sites** | |  |  |  |  |
| 1 | 86 (13.1%) | 216 (34.4%) | 75 (15.6%) | 477 (43.1%) | 854 (29.8%) |
| 2 | 200 (30.5%) | 273 (43.5%) | 136 (28.3%) | 421 (38.1%) | 1030 (35.9%) |
| 3 | 218 (33.3%) | 114 (18.2%) | 148 (30.8%) | 174 (15.7%) | 654 (22.8%) |
| 4 | 97 (14.8%) | 23 (3.7%) | 75 (15.6%) | 28 (2.5%) | 223 (7.8%) |
| 5+ | 54 (8.2%) | 2 (0.3%) | 46 (9.6%) | 6 (0.5%) | 108 (3.8%) |
| **Asian** |  |  |  |  |  |
| No | 641 (97.9%) | 551 (87.7%) | 367 (76.5%) | 963 (87.1%) | 2522 (87.9%) |
| Yes | 14 (2.1%) | 77 (12.3%) | 113 (23.5%) | 143 (12.9%) | 347 (12.1%) |
| **Albumin (g/L)** | |  |  |  |  |
| Mean (SD) | 38.8 (4.61) | 38.2 (5.51) | 38.5 (5.62) | 38.6 (5.57) | 38.5 (5.37) |
| Median [Min, Max] | 39.0 [21.0, 51.0] | 39.0 [10.0, 50.0] | 39.0 [15.8, 56.4] | 39.0 [16.7, 53.0] | 39.0 [10.0, 56.4] |
| Missing | 9 (1.4%) | 2 (0.3%) | 8 (1.7%) | 5 (0.5%) | 24 (0.8%) |
| **IC or TC >0** |  |  |  |  |  |
| Absent | 265 (40.5%) | 265 (42.2%) | 144 (30.0%) | 487 (44.0%) | 1161 (40.5%) |
| Present | 390 (59.5%) | 363 (57.8%) | 140 (29.2%) | 619 (56.0%) | 1512 (52.7%) |
| Missing | 0 (0%) | 0 (0%) | 196 (40.8%) | 0 (0%) | 196 (6.8%) |
| **Lactate dehydrogenase (U/L)** | |  |  |  |  |
| Mean (SD) | 319 (597) | 301 (328) | 299 (214) | 293 (209) | 302 (359) |
| Median [Min, Max] | 224 [2.17, 14600] | 220 [0, 5490] | 230 [0, 2040] | 230 [10.0, 2840] | 227 [0, 14600] |
| Missing | 17 (2.6%) | 11 (1.8%) | 20 (4.2%) | 12 (1.1%) | 60 (2.1%) |
| **Neutrophil-to-lymphocyte ratio** | |  |  |  |  |
| Mean (SD) | 5.09 (3.76) | 5.38 (4.38) | 4.89 (3.83) | 5.51 (7.02) | 5.28 (5.38) |
| Median [Min, Max] | 4.11 [0.923, 34.2] | 4.13 [0.259, 39.8] | 3.83 [0.770, 32.8] | 3.89 [0.0448, 97.0] | 3.98 [0.0448, 97.0] |
| Missing | 8 (1.2%) | 1 (0.2%) | 5 (1.0%) | 2 (0.2%) | 16 (0.6%) |
| **Liver Metastasis** | |  |  |  |  |
| No | 565 (86.3%) | 529 (84.2%) | 419 (87.3%) | 966 (87.3%) | 2479 (86.4%) |
| Yes | 90 (13.7%) | 99 (15.8%) | 61 (12.7%) | 140 (12.7%) | 390 (13.6%) |
| **Sum of longest diameter (mm)** | |  |  |  |  |
| Mean (SD) | 80.3 (49.6) | 97.1 (53.1) | 83.8 (55.2) | 79.2 (47.3) | 84.2 (51.0) |
| Median [Min, Max] | 70.0 [10.0, 392] | 90.0 [10.0, 309] | 71.0 [10.0, 339] | 70.0 [10.0, 299] | 75.0 [10.0, 392] |
| **Sex** |  |  |  |  |  |
| Female | 284 (43.4%) | 117 (18.6%) | 162 (33.8%) | 443 (40.1%) | 1006 (35.1%) |
| Male | 371 (56.6%) | 511 (81.4%) | 318 (66.3%) | 663 (59.9%) | 1863 (64.9%) |

Note: ECOG=Eastern Cooperative Oncology Group, IC=tumor-infiltrating immune cells; TC=tumor-infiltrating tumor cells; N: number of patients; min: minimum; max: maximum; SD: standard deviation.
