# Supplementary material for: External validation of a tumor growth inhibition-overall survival model in non-small-cell lung cancer based on atezolizumab studies using alectinib data
Source: Cancer Chemother Pharmacol. 2023 Jul 6;92(3):205–10. doi: 10.1007/s00280-023-04558-z (PMC10363035; doi:10.1007/s00280-023-04558-z)
Supplement: Supplementary file 5 — Supplementary file5 (DOCX 14 KB) [file 280_2023_4558_MOESM5_ESM.docx]

**Table S3** Parameter estimates from the updated and the historical TGI-OS models in atezolizumab NSCLC studies

|  | **Updated Model** | **Historical Model** |
| --- | --- | --- |
| **Parameter** | **Estimate** | **Estimate** |
| Intercept | 2.87 | 3.47 |
| logKG | -0.642 | -0.616 |
| Albumin (g/L) | 0.0262 | 0.0135 |
| ECOG PS (≥1 vs. 0) | -0.270 | -0.233 |
| Race (Asian vs. non-Asian) | 0.319 | 0.244 |
| Number of metastatic sites | -0.0733 | -0.0764 |
| Neutrophil-to-lymphocyte ratio | -0.0138 | -0.009 |
| Liver metastasis (yes vs. no) | -0.174 | -0.118 |
| Baseline SLD (mm) | -0.00122 |  |
| Line of therapy (2+ vs. 1) | -0.103 | -0.109 |
| Sex (Female vs. Male) | 0.0840 |  |
| C-reactive Protein (mg/L) |  | -0.00385 |
| IC or TC (>0 vs. 0) |  | 0.119 |
| Lactate dehydrogenase (U/L) |  | -0.00014 |
| Log(scale) | -0.225 | -0.264 |

Note: Survival time was analyzed in days. ECOG, Eastern Cooperative Oncology Group performance status (reference group is 0); log(KG), log of tumor growth rate constant (1/week) from the tumor growth inhibition model; p, Wald test p value; scale, standard deviation of log(OS); SE, standard error of parameter estimate; SLD, sum of longest diameter; IC: tumor-infiltrating immune cells; TC: tumor-infiltrating tumor cells (reference group is IC and TC = 0); z, Wald statistic.

* Number of metastatic sites was equal to 5 if the number is ≥ 5, otherwise it is equal to the number of metastatic sites
